# Supplementary material for: Tight junction protein occludin regulates progenitor Self-Renewal and survival in developing cortex
Source: eLife. 2019 Dec 3;8:e49376. doi: 10.7554/eLife.49376 (PMC6890460; doi:10.7554/eLife.49376)
Supplement: Supplementary file 1. — A compiled list of all primer sequences used in this study, cross-checked by experiment and figure number. [file elife-49376-supp1.docx]

| Experiment | Figure # | Primer Name | Primer Sequence | Species |
| --- | --- | --- | --- | --- |
| RT-PCR | 2 | Full-length 5’UTR Forward Primer | AGCCTGGACATTTTGCTC | Mouse |
| RT-PCR | 2 | ΔN 5’ UTR Forward Primer | ATAGCCATTGTCCTGGGGTT | Mouse |
| RT-PCR | 2 | Reverse primer spanning Ocln exons 4 and 5 | ACCCACTCTTCAACATTGGG | Mouse |
| CRISPR mutagenesis | 5 | Guide RNA sg#5 targeting exon 3 of OCLN | GCCTCTGTCCCAGGCAAGCGTGG | Human |
| CRISPR mutagenesis | 5 | Guide RNA sg#11 targeting exon 3 of OCLN | GGCTACCCTTATGGAGGAAGTGG | Human |
| CRISPR Off-target | 5 – Figure Suppl. 2 | Sg5 Mutant – Off-target loci #1 - Forward | ACAGGTGTGAGCTACTGTGC | Human |
| CRISPR Off-target | 5 – Figure Suppl. 2 | Sg5 Mutant – Off-target loci #1 - Reverse | TCACCTCACCATGCCATCAC | Human |
| CRISPR Off-target | 5 – Figure Suppl. 2 | Sg5 Mutant – Off-target loci #2 - Forward | GGCTTTGGGATGGGCTAAGT | Human |
| CRISPR Off-target | 5 – Figure Suppl. 2 | Sg5 Mutant – Off-target loci #2 - Reverse | TGGGTTGGTGCCAAGTCTTT | Human |
| CRISPR Off-target | 5 – Figure Suppl. 2 | Sg5 Mutant – Off-target loci #3 - Forward | TTCCCACAGTGCTCTCTCCT | Human |
| CRISPR Off-target | 5 – Figure Suppl. 2 | Sg5 Mutant – Off-target loci #3 - Reverse | ACATCTGAGGACATTGCCCG | Human |
| CRISPR Off-target | 5 – Figure Suppl. 2 | Sg5 Mutant – Off-taget Loci #4 - Forward | CAAGCACAACGTCTGGGTTC | Human |
| CRISPR Off-target | 5 – Figure Suppl. 2 | Sg5 Mutant – Off-target loci #4 - Reverse | TGGCTTGCCTTCTCTGACTT | Human |
| CRISPR Off-target | 5 – Figure Suppl. 2 | Sg5 Mutant – Off-taget Loci #5 - Forward | GTTTCAGCATGTTGCCCAGG | Human |
| CRISPR Off-target | 5 – Figure Suppl. 2 | Sg5 Mutant – Off-target loci #5 - Reverse | TGGTGACCAGCCAGATGAAC | Human |
| CRISPR Off-target | 5 – Figure Suppl. 2 | Sg5 Mutant – Off-taget Loci #6 - Forward | GCACTTCCAGCCTCCAGAAT | Human |
| CRISPR Off-target | 5 – Figure Suppl. 2 | Sg5 Mutant – Off-target loci #6 - Reverse | ACACAGACACATGCAGAGGG | Human |
| CRISPR Off-target | 5 – Figure Suppl. 2 | Sg11 Mutant – Off-taget Loci #1 - Forward | TAACCACCACTACCACCAGC | Human |
| CRISPR Off-target | 5 – Figure Suppl. 2 | Sg11 Mutant – Off-target loci #1- Reverse | GGAAGAACAAGCATCTTTCTCC | Human |
| CRISPR Off-target | 5 – Figure Suppl. 2 | Sg11 Mutant – Off-taget Loci #2 - Forward | TGGGTGAAAGTCCTGATGCC | Human |
| CRISPR Off-target | 5 – Figure Suppl. 2 | Sg11 Mutant – Off-target loci #2 - Reverse | TAAGGAAGTGGCTCAGCGTG | Human |
| Spheroid Characterization | 6 – Figure Suppl. 1 | OCT4 – Forward | TCTTCAGGAGATATGCAAAGCAG | Human |
| Spheroid Characterization | 6 – Figure Suppl. 1 | OCT4 – Reverse | CTGATCTGCTGCAGTGTGGG | Human |
| Spheroid Characterization | 6 – Figure Suppl. 1 | PAX6 - Forward | GTGTCCAACGGATGTGTGAG | Human |
| Spheroid Characterization | 6 – Figure Suppl. 1 | PAX6 - Reverse | CTAGCCAGGTTGCGAAGAAC | Human |
| Spheroid Characterization | 6 – Figure Suppl. 1 | TBR2 – Forward | GGTTCCAGGTTCTGGCTTCC | Human |
| Spheroid Characterization | 6 – Figure Suppl. 1 | TBR2 – Reverse | AAAGGAAACATGCGCCTGCC | Human |
| Spheroid Characterization | 6 – Figure Suppl. 1 | FOXG1 – Forward | AGGAGGGCGAGAAGAAGAAC | Human |
| Spheroid Characterization | 6 – Figure Suppl. 1 | FOXG1 – Reverse | TCACGAAGCACTTGTTGAGG | Human |
| Spheroid Characterization | 6 – Figure Suppl. 1 | OTX1 – Forward | GCCTCCCCTTCCAGTCTTTC | Human |
| Spheroid Characterization | 6 – Figure Suppl. 1 | OTX1 – Reverse | GGGCAGAAACACGCCAGTTA | Human |
| Spheroid Characterization | 6 – Figure Suppl. 1 | DLX1 – Forward | CGCTTCAATGGCAAGGGAAA | Human |
| Spheroid Characterization | 6 – Figure Suppl. 1 | DLX1 – Reverse | ACCAGATCTTGACCTGAGTCT | Human |
| Spheroid Characterization | 6 – Figure Suppl. 1 | NKX2-1 – Forward | CTCGCTCGCTCATTTGTTGG | Human |
| Spheroid Characterization | 6 – Figure Suppl. 1 | NKX2-1 – Reverse | TCGGCGGCGGCTGAG | Human |
| Spheroid Characterization | 6 – Figure Suppl. 1 | GAPDH – Forward | GGATTTGGTCGTATTGGG | Human |
| Spheroid Characterization | 6 – Figure Suppl. 1 | GAPDH - Reverse | GGAAGATGGTGATGGGATT | Human |
